# FAM83H and Nectin1 expression are related with survival and relapse of bladder urothelial carcinoma patients

**DOI:** 10.1186/s12894-021-00908-2

**Published:** 2021-10-08

**Authors:** Ae-Ri Ahn, Sang Jae Noh, Usama Khamis Hussein, Ho Sung Park, Myoung Ja Chung, Ho Lee, Woo Sung Moon, Myoung Jae Kang, Hyung Jin Kim, Na Ri Lee, Kyu Yun Jang, Kyoung Min Kim

**Affiliations:** 1 Department of Pathology, Jeonbuk National University Medical School, Jeonju, Republic of Korea; 2 Department of Forensic Medicine, Jeonbuk National University Medical School, Jeonju, Republic of Korea; 3 Faculty of Science, Beni-Suef University, Beni-Suef, Egypt; 4 Department of Urology, Jeonbuk National University Medical School, Jeonju, Republic of Korea; 5 Department of Internal Medicine, Jeonbuk National University Hospital-Jeonbuk National University Medical School, Jeonju, Republic of Korea; 6 Research Institute of Clinical Medicine of Jeonbuk National University-Biomedical Research Institute of Jeonbuk National University Hospital, Jeonju, Republic of Korea

## Abstract

**Background:**

FAM83H was originally reported to be essential for dental enamel formation. However, FAM83H has recently been implicated in tumorigenesis and tumor progression. Analysis of a publicly available gene expression database revealed a significant correlation between FAM83H and Nectin1 mRNA expression and bladder urothelial carcinoma (BUC). Therefore, we investigated the association between FAM83H and Nectin1 expression levels and the survival and recurrence of BUC in BUC patients using a tissue microarray.

**Methods:**

We performed immunohistochemical staining of FAM83H and Nectin1 in 165 human BUC tissue sections, and analyzed the prognostic significance of FAM83H and Nectin1 expression.

**Results:**

Both FAM83H and Nectin1 were mainly expressed in the cytoplasm, and their expression was significantly associated. FAM83H expression was significantly correlated with higher histologic grade, higher T stage, higher TNM stage, and recurrence. Nectin1 expression was significantly associated with higher histologic grade and recurrence. Univariate analysis showed FAM83H expression and Nectin1 expression were significantly associated with worse overall survival (OS) and shorter relapse-free survival (RFS) of BUC patients. In multivariate analysis, levels of FAM83H and Nectin1 were independent indicators of shorter survival of BUC patients.

**Conclusions:**

Our results suggest that FAM83H and Nectin1 are important in the progression of BUC, and that expression patterns of these two proteins can be used as prognostic indicators of survival in BUC patients.

**Supplementary Information:**

The online version contains supplementary material available at 10.1186/s12894-021-00908-2.

## Introduction

Bladder cancer is a common cancer, accounting for approximately 3.0% of new cancers and 2.1% of cancer deaths worldwide [1]. The prognosis of patients with bladder cancer is relatively good, with a 5-year survival rate of approximately 70% [2]. However, the survival rate has not improved over the years. Moreover, the prognosis of patients with metastasis is poor, with a median survival of 5–7 months and a 5-year survival rate of only 15% [3, 4]. Therefore, new approaches are necessary to supplement the current platinum-based therapeutic strategy.

There are two distinct pathways for development of bladder urothelial carcinoma (BUC): a hyperplasia pathway and a dysplasia pathway [5–8]. The hyperplasia pathway is more common and accounts for 80% of BUCs [6]. Tumors that develop by activation of the hyperplasia pathway first manifest as urothelial hyperplasia with advancement to low-grade papillary urothelial carcinoma [6, 7, 9]. The hyperplasia pathway is characterized by alterations in the *FGFR3* gene, and is genetically stable [8]. Tumors that develop due to activation of this pathway are non-aggressive [9]. Tumors that result from activation of the dysplasia pathway initially present as dysplasia that then progresses to high-grade papillary urothelial carcinoma or urothelial carcinoma in situ and account for approximately 20% of BUC cases. These tumors are associated with a high risk of muscle invasion and metastasis [7, 8]. This pathway is genetically instable and inactivating mutations of *TP53* are the most common genetic alterations [7].

FAM83H was originally reported to be an essential molecule for dental enamel formation [10, 11]. However, FAM83H has more recently been found to be involved in tumorigenesis and tumor progression [12–15]. In colorectal cancer, FAM83H regulates the organization of the keratin cytoskeleton and formation of desmosomes and is involved in the movement of cancer cells [12]. FAM83H has also been suggested to contribute to the progression of androgen-independent prostate cancer [16]. FAM83H is transcriptionally controlled by the well-known oncogene MYC and regulates the proliferation of hepatocellular carcinoma cells [13]. In osteosarcomas, FAM83H stabilizes β-catenin and regulates Wnt signaling [14]. Expression of FAM83H was found to be associated with poor survival of renal cell carcinoma patients [17] and FAM83H expression was associated with a worse prognosis and found to be involved in PI3K-Akt-mTOR signaling in pancreatic cancer [18]. However, neither the expression nor role of FAM83H in BUC has been studied to date.

Nectin1 is a member of the Nectin family of immunoglobulin-like cell adhesion molecules. Nectins participate in various cellular activities such as cell differentiation, polarization, migration, proliferation, and survival [19, 20]. Although limited number of studies have analyzed the role of Nectin1 in tumors, most of these studies have found that Nectin1 expression is associated with cancer progression. In colorectal cancer, Nectin1 expression was associated with a worse 3-year progression-free survival rate [21]. Furthermore, Nectin1 expression in cancer-associated fibroblasts was correlated with a poor prognosis in pancreatic ductal adenocarcinoma patients [22]. However, similar to FAM83H, the role of Nectin1 in BUC has not previously been explored.

We analyzed the BUC dataset of The Cancer Genome Atlas (TCGA), Cell 2017 [23] through the cBioPortal public database (http://www.cbioportal.org), and found that Nectin1 mRNA expression was significant associated with FAM83H mRNA expression in BUCs (Additional file 1: Figure 1). Therefore, in this study, we investigated the prognostic impact of FAM83H and Nectin1 expression using immunohistochemical staining of a human BUC tissue microarray. In addition, we also evaluated FAM83H and Nectin1 expression in low-grade and high-grade non-invasive BUCs because of the considerable differences in biologic behavior and underlying genetic alterations of these BUCs.

## Materials and methods

### Ethical approval

This study was approved by the Institutional Review Board of Jeonbuk National University Hospital (IRB number, CUH 2020-02-007) and was performed in accordance with the principles of the Declaration of Helsinki. Signed informed consent form was obtained from all eligible participants.

### Patients and follow-up

One hundred sixty-five BUC patients who underwent surgery at Jeonbuk National University Hospital between January 2008 and September 2018 were included in this study. Clinicopathologic information was obtained by reviewing medical records. Clinicopathological factors evaluated in this study were sex, age, histologic grade, T stage, N stage, M stage, TNM stage, and recurrence. Histologic slides were reviewed according to the WHO classification of tumors of the urinary system and male genital organs [24]. The 8th edition of the American Joint Committee Cancer Staging System was referenced to classify the TNM stage of BUC patients [3].

After the surgery, follow-up of the patients was carried out every 3 months in the first year. After the first year, the patients visited the hospital every 6 to 8 months period. During the follow-up, physical examination and blood and urine analysis were performed. For 3 years after the surgery, cystoscopic screening was performed in every 6 months. The survival data were most recently renewed in March 2020. The mean follow-up duration was 53.8 months.

### Immunohistochemical staining and scoring

In May 2020, we established a tissue microarray from paraffin-embedded tissue blocks of surgical specimens of BUC patients to evaluate the immunohistochemical expression of FAM83H and Nectin1. Two 3.0 mm cores without necrosis or degenerative changes were obtained from the tumor for each case. Tissue sections were deparaffinized followed by antigen retrieval using a microwave oven in pH 6.0 antigen retrieval solution (DAKO, Glostrup, Denmark). Then, tissue sections were incubated with primary antibodies for FAM83H (1:100, catalogue no. A304-328A, Bethyl Laboratories, Montgomery, TX) and Nectin1 (1:50, catalogue no. sc-21722, Santa Cruz Biotechnology, Santa Cruz, CA) overnight at 4ºC.

Immunohistochemical staining results were evaluated by two pathologists (KYJ and KMK) by consensus without knowledge of the patients’ clinical status using a multi-viewing microscope (Nikon Eclipse 80i; Nikon, Tokyo, Japan). Both FAM83H and Nectin1 were mainly expressed in the cytoplasm. Intensity of immunohistochemical staining was scored as follows: negative, 0; weak, 1; intermediate, 2; and strong, 3. In addition, the proportion of tissue area stained was scored as follows: no staining, 0; ~ 1% staining, 1; 2–10% staining, 2; 11–33% staining, 3; 34–66% staining, 4; and 67–100% staining, 5. The staining intensity score and staining area score were added for each tissue section, and then the scores for the two tissue sections per case were added to obtain the final score for each patient. The immunohistochemical staining score therefore ranged from 0 to 16.

### Statistical analysis

Patients were divided into negative and positive subgroups based on the immunohistochemical expression of FAM83H and Nectin1. The cut-off points for both FAM83H and Nectin1 were determined by receiver operating characteristic curve analysis as the highest predictive point for death [25]. We excluded non-invasive low-grade BUC cases from the survival analysis because of the significant difference in the prognosis of these cases compared to non-invasive high-grade and invasive cases. Overall survival (OS) and relapse-free survival (RFS) were evaluated through March 2020. In OS analysis, death of the patient by urothelial carcinoma was considered an event. Cases characterized by death due to other causes or being alive at the last follow-up were censored. Relapse of urothelial carcinoma or patient death by urothelial carcinoma were treated as events in the RFS analysis. Cox proportional hazards regression analysis and Kaplan–Meier survival analysis were utilized to evaluate the prognosis of urothelial carcinoma patients. Features independently associated with survival were included in multivariate analysis of Cox’s proportional hazards model using the stepwise method. The relationship between immunohistochemical expression and clinicopathological factors was analyzed with Pearson’s chi-square test. Pearson’s method was utilized to evaluated the correlation between FAM83H and Nectin1 expression. SPSS software (IBM, version 19.0, Armonk, NY) was used throughout for statistical analysis. P values less than 0.05 were considered statistically significant.

## Results

### Expression of FAM83H and Nectin1 in BUC tissue sections and the correlation between expression levels of these two proteins and clinicopathologic characteristics

**Fig. 1:**
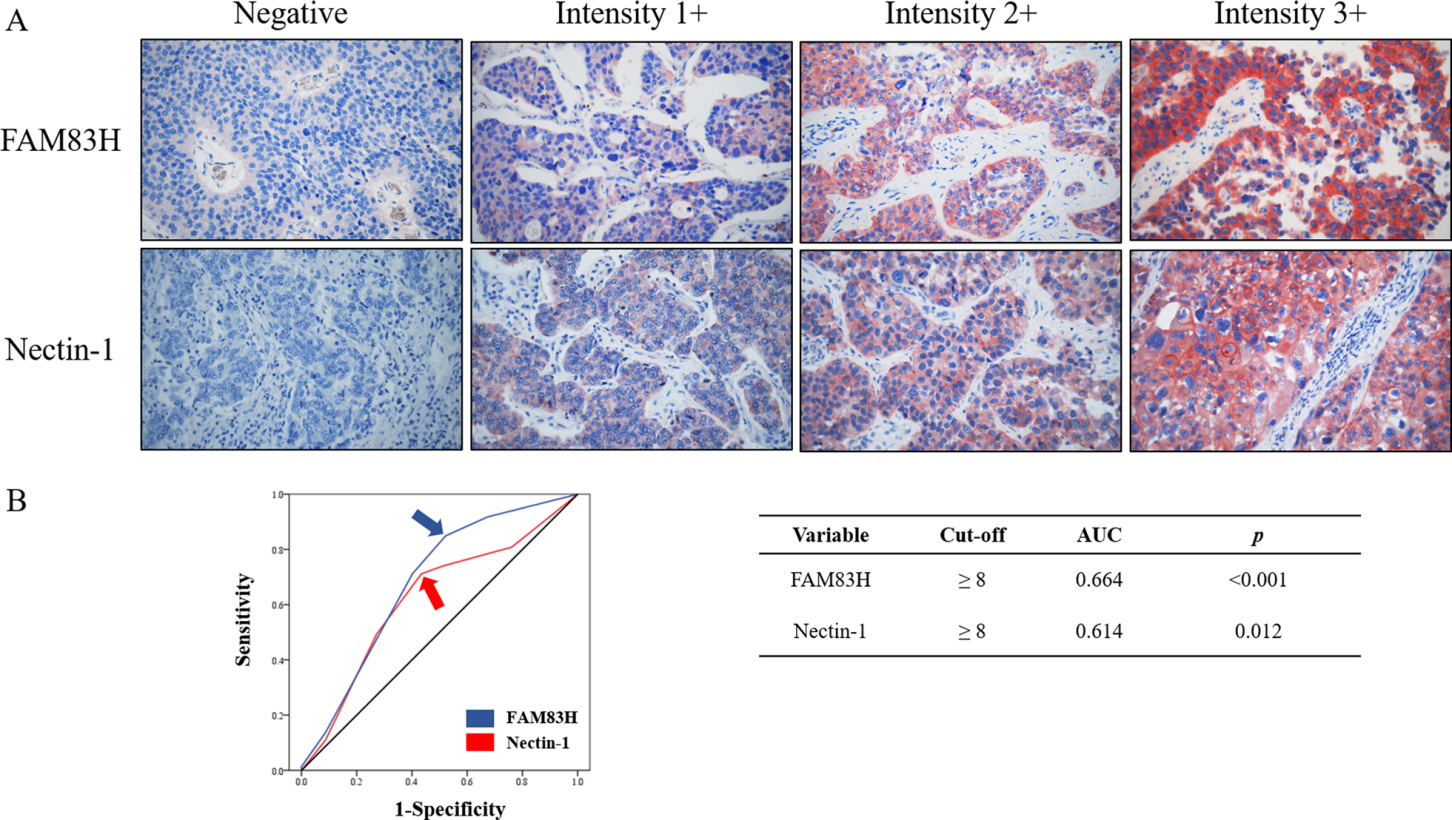
Immunohistochemical expression of FAM83H and Nectin1 in Bladder urothelial carcinoma. **A** FAM83H and Nectin1 are expressed mainly in the cytoplasm of the cancer cells. Original magnification: × 400. **B** Receiver operating characteristic curve analysis to determine cut-off points for the expression of nuclear FAM83H (blue arrow) and cytoplasmic Nectin1 (red arrow). The cut-off points indicate the point of the highest area under the curve (AUC) to predict the death of bladder urothelial carcinoma patients

Typical immunohistochemical staining results for FAM83H and Nectin1 in BUC tissue sections are shown in Fig. 1. Both FAM83H and Nectin1 were expressed primarily in the cytoplasm (Fig. 1A). We performed receiver operating characteristic curve analysis according to the death of BUC patients to assign patients to FAM83H and Nectin1 negative- and positive expression groups. The cut-off points for the expression of FAM83H and Nectin1 were both eight (Fig. 1B). Using these cut-off values, 110 (66.7%) and 101 (61.2%) BUC patients were classified as belonging to the FAM83H positive-group and Nectin1 positive-group, respectively.

Positive FAM83H expression was significantly correlated with higher histologic grade (*P* < 0.001), higher T stage (*P* = 0.004), higher TNM stage (*P* = 0.008), and recurrence (*P* = 0.022*)* (Table 1). Positive Nectin1 expression was significantly associated with higher histologic grade (*P* < 0.001), higher N stage (*P* = 0.031), and recurrence (*P* = 0.014) (Table 1).

**Table 1:** Clinicopathologic variables and the expression of FAM83H and Nectin1 in bladder urothelial carcinomas

Characteristics		Total	FAM83H expression		*p*	Nectin1 expression		*p*	Combined expression			*p*
			Positive	Negative		Positive	Negative		FAM83H+/Nectin1+	FAM83H+/Nectin1− or FAM83H−/Nectin1+	FAM83H−/Nectin1−	
All cases		165	110 (66.7%)	55 (33.3%)		101 (61.2%)	64 (38.8%)		86 (52.1%)	39 (23.6%)	40 (24.2%)	
Sex	Male	145	96 (66.2%)	49 (33.8%)		87 (60%)	58 (40%)		74 (51%)	35 (24.1%)	36 (24.8%)	
	Female	20	14 (70%)	6 (30%)	0.736	14 (70%)	6 (30%)	0.39	12 (60%)	4 (20%)	4 (20%)	0.753
Age (years)	≤ 65	44	25 (56.8%)	19 (43.2%)		25 (56.8%)	19 (43.2%)		20 (45.5%)	10 (22.7%)	14 (31.8%)	
	> 65	121	85 (70.2%)	36 (29.8%)	0.106	76 (62.8%)	45 (37.2%)	0.485	66 (54.5%)	29 (24%)	26 (21.5%)	0.377
Histologic grade	Low	56	23 (41.1%)	33 (58.9%)		21 (37.5%)	35 (62.5%)		14 (25%)	16 (28.6%)	26 (46.4%)	
	High	109	87 (79.8%)	22 (20.2%)	< 0.001	80 (73.4%)	29 (26.6%)	< 0.001	72 (66.1%)	23 (21.1%)	14 (12.8%)	< 0.001
T stage	Ta	37	18 (48.6%)	19 (51.4%)		19 (51.4%)	18 (48.6%)		12 (32.4%)	13 (35.1%)	12 (32.4%)	
	T1	77	50 (64.9%)	27 (35.1%)		46 (59.7%)	31 (40.3%)		39 (50.6%)	18 (23.4%)	20 (26%)	
	T2-4	51	42 (82.4%)	9 (17.6%)	0.004	36 (70.6%)	15 (29.4%)	0.176	35 (68.6%)	8 (15.7%)	8 (15.7%)	0.021
N stage	N0	158	104 (65.8%)	54 (34.2%)		94 (59.5%)	64 (40.5%)		80 (50.6%)	38 (24.1%)	40 (25.3%)	
	N1-3	7	6 (85.7%)	1 (14.3%)	0.275	7 (100%)	0 (0%)	0.031	6 (85.7%)	1 (14.3%)	0 (0%)	0.163
M stage	M0	160	105 (65.6%)	55 (34.4%)		97 (60.6%)	63 (39.4%)		82 (51.3%)	38 (23.8%)	40 (25%)	
	M1	5	5 (100%)	0 (0%)	0.108	4 (80%)	1 (20%)	0.381	4 (80%)	1 (20%)	0 (0%)	0.359
TNM stage	Stage 0	37	18 (48.6%)	19 (51.4%)		19 (51.4%)	18 (48.6%)		12 (32.4%)	13 (35.1%)	12 (32.4%)	
	Stage I	77	50 (64.9%)	27 (35.1%)		46 (59.7%)	31 (40.3%)		39 (50.6%)	18 (23.4%)	20 (26%)	
	Stage II, III	46	37 (80.4%)	9 (19.6%)		32 (69.6%)	14 (30.4%)		31 (67.4%)	7 (15.2%)	8 (17.4%)	
	Stage IV	5	5 (100%)	0 (0%)	0.008	4 (80%)	1 (20%)	0.298	4 (80%)	1 (20%)	0 (0%)	0.056
Recurrence	Present	49	39 (79.6%)	10 (20.4%)		37 (75.5%)	12 (24.5%)		30 (61.2%)	16 (32.7%)	3 (6.1%)	
	Absent	116	71 (61.2%)	45 (38.8%)	0.022	64 (55.2%)	52 (44.8%)	0.014	56 (48.3%)	23 (19.8%)	37 (31.9%)	0.002

### Expression of FAM83H and Nectin1 in non-invasive BUCs

Because of the significant difference in prognosis and underlying molecular characteristics of low- and high-grade non-invasive BUCs, we investigated FAM83H and Nectin1 expression according to histologic grade in non-invasive BUCs (Table 2). High-grade non-invasive BUC tissue sections were significantly more likely to be positive for FAM83H and Nectin1 expression than low-grade non-invasive BUCs (*P* = 0.02, *P* = 0.034, respectively).

**Table 2:** Expression of FAM83H and Nectin1 in non-invasive bladder urothelial carcinomas

Characteristics		Total	FAM83H expression		*p*	Nectin1 expression		*p*	Combined expression			*p*
			Positive	Negative		Positive	Negative		FAM83H+/Nectin1+	FAM83H+/Nectin1− or FAM83H−/Nectin1+	FAM83H−/Nectin1−	
Histologic grade	Low	27	10 (37%)	17 (63%)		11 (40.7%)	16 (59.3%)		6 (22.2%)	9 (33.3%)	12 (44.4%)	
	High	10	8 (80%)	2 (20%)	0.02	8 (80%)	2 (20%)	0.034	6 (60%)	4 (40%)	0 (0%)	0.021

### Expression of FAM83H and Nectin1 correlates with poor prognosis in BUC patients

**Fig. 2:**
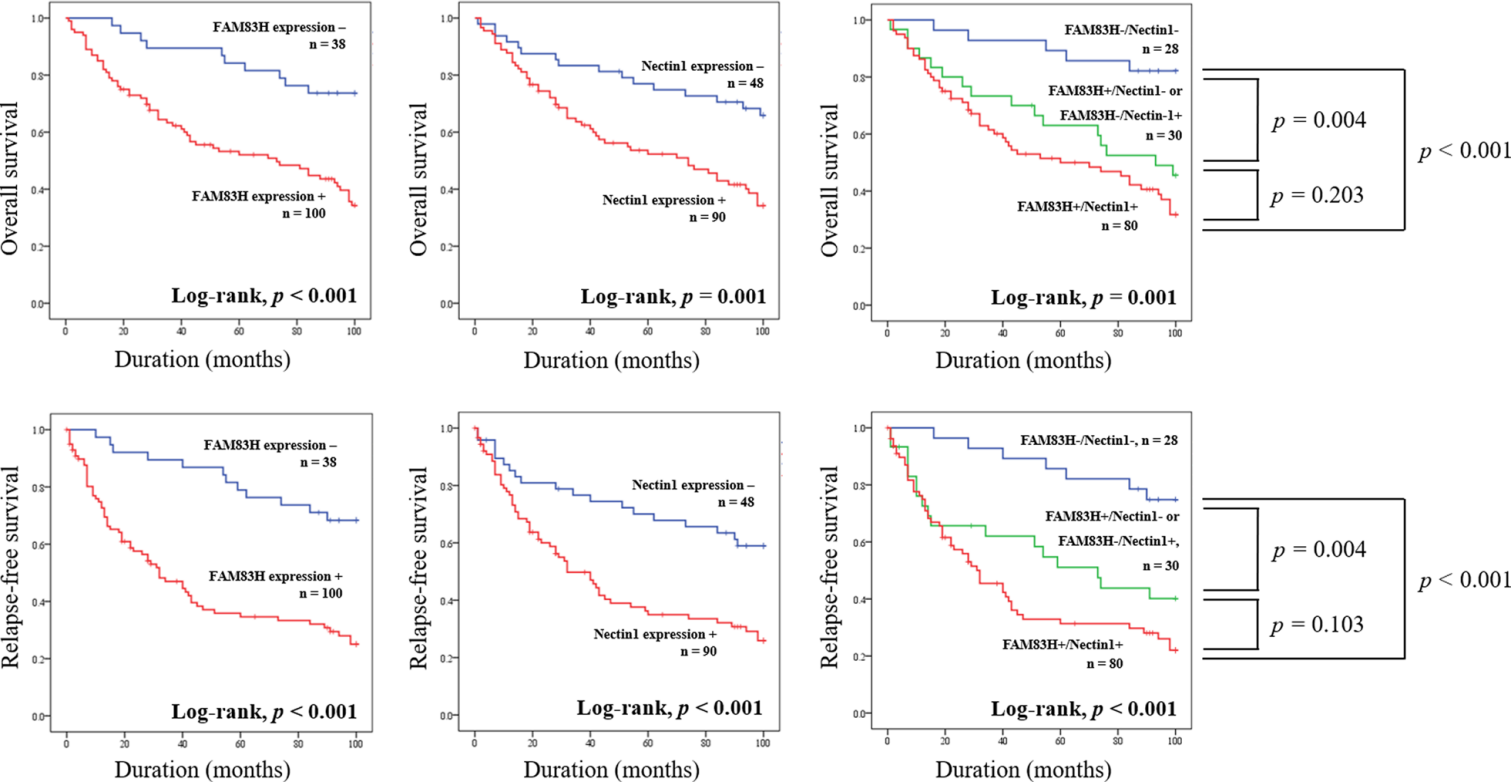
Survival analysis according to expression of FAM83H and Nectin1 in bladder urothelial carcinoma patients. Kaplan–Meier survival curves for overall survival and relapse-free survival of bladder urothelial carcinoma patients according to the individual and coexpression of FAM83H, and Nectin1

We excluded non-invasive low-grade BUCs from the survival analysis because these tumors rarely progress to invasive carcinoma. Univariate analysis showed that histologic grade, T stage, N stage, M stage, TNM stage, FAM83H expression (*P* < 0.001), and Nectin1 expression (*P* = 0.002) were significantly associated with the OS of bladder urothelial carcinoma patients (Table 3). Histologic grade, T stage, N stage, TNM stage, FAM83H expression (*P* < 0.001), and Nectin1 expression (*P* = 0.001) were significantly correlated with the RFS of BUC patients based on univariate analysis (Table 3). FAM83H-positive patients had a 3.42-fold [95% confidence interval (95% CI); 1.75–6.7, *P* < 0.001] increased risk of death and a 3.83-fold (95% CI 2.06–7.12, *P* < 0.001) increased risk of relapse or death compared to FAM83H-negative patients (Table 3). Nectin1-positive patients had a 2.48-fold (95% CI 1.42–4.35, *P* = 0.002) increased risk of death and a 2.48-fold (95% CI 1.47–4.17, *P* = 0.001) increased risk of relapse or death compared to Nectin1-negative patients (Table 3). Kaplan–Meier survival analysis curves for OS and RFS of BUC patients according to the expression of FAM83H and Nectin1 are presented in Fig. 2.

**Table 3:** Univariate Cox proportional hazards regression analysis for overall survival and relapse-free survival in non-invasive high-grade and invasive bladder urothelial carcinomas

Characteristics	OS		RFS	
	HR (95% CI)	*p*	HR (95% CI)	*p*
Sex, female (vs. male)	1.014 (0.503–2.042)	0.969	1.065 (0.532–2.134)	0.859
Age, y ≥ 65 (vs. < 65)	1.776 (0.953–3.309)	0.055	1.265 (0.738–2.167)	0.392
Grade, high (vs. low)	2.924 (1.398–6.117)	0.004	2.547 (1.307–4.963)	0.006
T stage, Ta	1	< 0.001	1	0.012
T1	1.372 (0.419–4.497)	0.601	0.916 (0.361–2.323)	0.854
T2–4	5.054 (1.55–16.475)	0.007	1.842 (0.717–4.729)	0.204
N stage, N1-3 (vs. N0)	5.832 (2.575–13.211)	< 0.001	3.087 (1.319–7.227)	0.009
M stage, M1 (vs. M0)	7.814 (3.038–20.096)	< 0.001	0.894 (0.123–6.48)	0.912
TNM stage, Stage 0	1	< 0.001	1	0.027
Stage I	1.374 (0.419–4.503)	0.6	0.916 (0.362–2.323)	0.916
Stage II, III	4.625 (1.41–15.177)	0.012	1.88 (0.731–4.835)	0.19
Stage IV	17.793 (4.119–76.872)	< 0.001	1.085 (0.126–9.368)	0.879
FAM83H, positive (vs. negative)	3.423 (1.748–6.704)	< 0.001	3.83 (2.06–7.121)	< 0.001
Nectin1, positive (vs. negative)	2.48 (1.415–4.346)	0.002	2.475 (1.469–4.169)	0.001
Combined expression, FAM83H−/Nectin1−	1	0.001	1	< 0.001
FAM83H−/Nectin1+ or FAM83H+/Nectin1−	3.89 (1.424–10.624)	0.008	3.36 (1.392–8.11)	0.007
FAM83H+/Nectin1+	5.579 (2.216–14.046)	< 0.001	5.309 (2.402–11.735)	< 0.001

We also performed multivariate analysis of OS and RFS in BUC patients. Factors significantly associated with OS or RFS were included in the analysis. T stage, N stage, M stage, and FAM83H expression were independent prognostic factors associated with OS in BUC patients (Table 4, model 1). Positive-FAM83H expression group had a 3.14-fold (95% CI 1.59–6.22, *P* = 0.001) increased risk of death comparted to the negative-FAM83H expression group (Table 4, model 1). TNM stage and FAM83H expression were independent prognostic factors of RFS based on multivariate analysis (Table 5, model 1). FAM83H-positive expression group had a 3.69-fold (95% CI 1.98–6.89, *P* < 0.001) increased risk of relapse or death compared to the FAM83H-negative expression group (Table 5, model 1).

**Table 4:** Multivariate Cox regression analysis for overall survival in non-invasive high-grade and invasive bladder urothelial carcinomas

Characteristics	OS	
	HR (95% CI)	*p*
Model 1^a^		
T stage, Ta	1	< 0.001
T1	1.647 (0.501–5.411)	0.411
T2-4	4.78 (1.434–15.932)	0.011
N stage, N1-3 (vs. N0)	2.774 (1.185–6.492)	0.019
M stage, M1 (vs. M0)	3.435 (1.291–9.142)	0.013
FAM83H positive (vs. negative)	3.141 (1.587–6.215)	0.001
Model 2^b^		
T stage, Ta	1	< 0.001
T1	1.831 (0.556–6.025)	0.32
T2-4	5.76 (1.707–19.436)	0.005
N stage, N1-3 (vs. N0)	2.382 (1.016–5.586)	0.046
M stage, M1 (vs. M0)	3.428 (1.277–9.196)	0.014
Combined expression, FAM83H−/Nectin1−	1	0.002
FAM83H−/Nectin1+ or FAM83H+/Nectin1−	4.731 (1.7–13.163)	0.003
FAM83H+/Nectin1+	5.46 (2.144–13.905)	< 0.001

*OS* overall survival, *HR* hazard ratio, *95% CI* 95% confidence interval

^a^Variables considered in model 1 were histologic grade, T stage, N stage, M stage, TNM stage, FAM83H expression, and Nectin1 expression

^b^Variables considered in model 2 were histologic grade, T stage, N stage, M stage, TNM stage, and combined expression of FAM83H and Nectin1

**Table 5:** Multivariate Cox regression analysis for relapse-free survival in non-invasive high-grade and invasive bladder urothelial carcinomas

	RFS	
	HR (95% CI)	*p*
Model 1^a^		
TNM stage, Stage 0	1	0.067
Stage I	1.075 (0.423–2.729)	0.88
Stage II, III	1.983 (0.77–5.106)	0.156
Stage IV	0.906 (0.105–7.837)	0.929
FAM83H positive (vs. negative)	3.691 (1.977–6.889)	< 0.001
Model 2^b^		
TNM stage, Stage 0	1	0.037
Stage I	1.175 (0.871–3.021)	0.738
Stage II, III	2.305 (0.871–6.1)	0.093
Stage IV	1.106 (0.128–9.584)	0.927
Combined expression, FAM83H−/Nectin1−	1	< 0.001
FAM83H−/Nectin1+ or FAM83H+/Nectin1−	3.877 (1.582–9.502)	0.003
FAM83H+/Nectin1+	5.272 (2.377–11.693)	< 0.001

*RFS* relapse free survival, *HR* hazard ratio, *95% CI* 95% confidence interval

^a^Variables considered in model 1 were histologic grade, T stage, N stage, TNM stage, FAM83H expression, and Nectin1 expression

^b^Variables considered in model 2 were histologic grade, T stage, N stage, TNM stage, and combined expression of FAM83H and Nectin1

### Co-expression patterns of FAM83H and Nectin1 expression and their correlations with clinicopathologic features and survival in patients with non-invasive high-grade or invasive BUC

We re-classified patients into three sub-groups (FAM83H+/Nectin1+, FAM83H+/Nectin1− or FAM83H−/Nectin1+, and FAM83H−/Nectin1−) based on FAM83H and Nectin1 expression. Co-expression of FAM83H/Nectin1 was significantly associated with histologic grade (*P* < 0.001), T stage (*P* = 0.021), and recurrence (*P* = 0.002) (Table 1). FAM83H/Nectin1 co-expression also showed a significant correlation with histologic grade in non-invasive BUCs (Table 2). The number of FAM83H+/Nectin1+ group was highest in high-grade non-invasive BUC with FAM83H−/Nectin1− group showing lowest number in high-grade non-invasive BUC (*P* = 0.021).

Univariate analysis indicated that co-expression of FAM83H/Nectin1 was significantly associated with the OS and RFS of BUC patients (Table 3). FAM83H+/Nectin1− and FAM83H−/Nectin1+ cases had a 3.89-fold (95% CI 1.42–10.62) and 3.36 (95% CI 1.39–8.11) greater risk of death and relapse or death, respectively, than FAM83H−/Nectin1− cases (Table 3). Co-expression of FAM83H+/Nectin1+ was associated with a 5.58-fold (95% CI 2.22–14.05) and 5.31 (95% CI 2.4–11.74) greater risk of death and relapse or death, respectively, compared to FAM83H−/Nectin1− cases (Table 3). Kaplan–Meier survival analysis curves for OS and RFS of BUC patients according to co-expression patterns of FAM83H and Nectin1 are presented in Fig. 2. Overall survival and relapse-free survival of BUC patients showed a step-wise decrease from the FAM83H−/Nectin1− group to the FAM83H+/Nectin1+ group. However, there was no significant differences in survival rate between the FAM83H+/Nectin1− and FAM83H−/Nectin1+ group versus the FAM83H+/Nectin1+ group (Fig. 2).

In multivariate analysis, co-expression pattern of FAM83H and Nectin1 was an independent prognostic factor. FAM83H+/Nectin1− and FAM83H−/Nectin1+ cases had a 4.73-fold (95% CI 1.7–13.16) greater risk of death than FAM83H−/Nectin1− cases while FAM83H+/Nectin1+ cases had a 5.46-fold (95% CI 2.14–13.91) greater risk of death than FAM83H−/Nectin1− cases (Table 4, model 2). FAM83H+/Nectin1− cases and FAM83H−/Nectin1+ cases had a 3.88-fold (95% CI 1.58–9.5) greater risk of death or relapse than FAM83H−/Nectin1− cases while FAM83H+/Nectin1+ cases had a 5.27-fold (95% CI 2.38–11.69) greater risk of death or relapse than FAM83H−/Nectin1− cases (Table 5, model 2).

### FAM83H and Nectin1 expression are significantly correlated

**Fig. 3:**
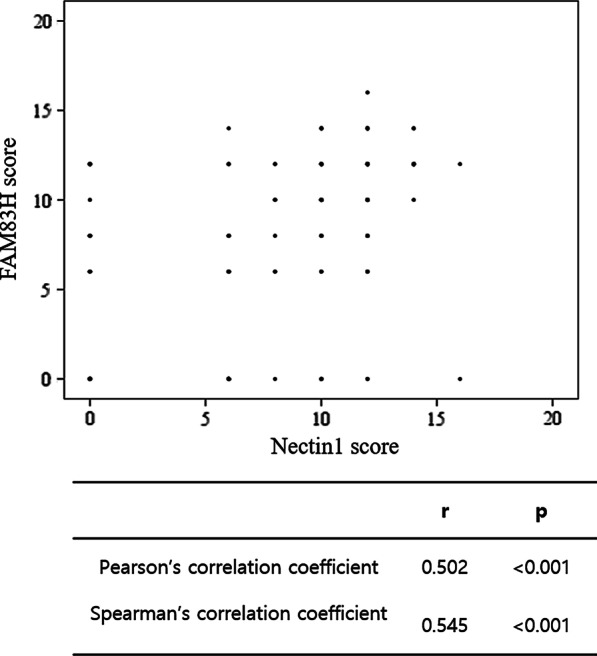
Scattergram comparing immunohistochemical score of FAM83H and Nectin1 expression in bladder urothelial carcinoma. Immunohistochemical score of FAM83H and Nectin1 showing significant positive correlation

Analysis of data in the cBioPortal database revealed that mRNA levels of FAM83H and Nectin1 were significantly correlated (Supplemental Figure S1). Therefore, we analyzed the relationship between immunohistochemical expression of FAM83H and Nectin1. The χ^2^ test showed significant associations between positive- and negative-expression groups of FAM83H and Nectin1 (*P* < 0.001) (Table 6). Moreover, there was a significant correlation between immunohistochemical staining scores for FAM83H and Nectin1 (both variables analyzed as continuous data, Pearson’s r = 0.502, *P* < 0.001; Spearman’s r = 0.545, *P* < 0.001; Fig. 3).

**Table 6:** Correlation between expression of FAM83H and Necin-1

Characteristics		FAM83H expression		*p*
		Positive	Negative	
Nectin1 expression	Positive	96 (58.2%)	16 (9.7%)	
	Negative	14 (8.5%)	39 (23.6%)	< 0.001

## Discussion

In the present study, we investigated the immunohistochemical expression of FAM83H and Nectin1 in a human BUC tissue microarray. This is the first report to assess if the expression of FAM83H and Nectin1 in BUCs is correlated. We found that (1) positive expression of FAM83H and Nectin1 was correlated with unfavorable clinicopathologic characteristics; (2) FAM83H and Nectin1 expression were significantly higher in high-grade non-invasive BUCs than low-grade BUCs; (3) BUC patients whose tumors were positive for FAM83H and Nectin1 had shorter OS and RFS; (4) individual and co-expression patterns of FAM83H and Nectin1 were poor independent prognostic factors for OS and RFS; and (5) expression of FAM83H and Nectin1 were significantly positively correlated.

Although FAM83H was originally identified as important in dental enamel formation, expression of this protein has been found to be increased in stomach, pancreas, liver, ovary, colon and breast cancers [26]. FAM83H is thought to stabilize β-catenin in osteosarcomas [14] while in colon cancer, FAM83H has been reported to contribute to tumor progression via keratin cytoskeleton disorganization [15]. Moreover, FAM83H is thought to be involved in hepatocellular carcinoma progression by controlling the transcription of MYC [13]. In addition, higher expression of FAM83H was found to be associated with shorter survival in patients with clear cell renal cell carcinoma, pancreatic cancer, or uterine cancer [17, 18, 26]. However in contrast to the majority of the reports, one study reported that FAM83H expression was associated with better disease-free survival in brain astrocytoma and oligodendroglioma patients [26]. Therefore, the role of FAM83H in tumorigenesis or tumor progression may differ according to cancer type. Consistent with the majority of reports, we found that FAM83H expression was associated with tumor progression in BUC patients. Positive expression of FAM83H was associated with higher histologic grade, higher T stage, and higher TNM stage BUCs. Moreover, patients with positive FAM83H expression had significantly shorter OS and RFS in univariate analysis. Multivariate analysis revealed that FAM83H expression was an independent factor for shorter OS and RFS in BUC patients.

Nectin proteins are Ca^2+^-independent immunoglobulin-like cell adhesion molecules involved in cell to cell adhesion, and are involved in various cellular activities such as differentiation, proliferation, survival, and movement [19]. Nectin1 is one of the four members of the Nectin family and is expressed in many cell types, including neurons, fibroblasts, and epithelial cells [27]. In the context of cancer research, one study reported that Nectin1 expression was associated with shorter progression-free survival of colorectal cancer patients [21]. Another study reported that Nectin1 expression in cancer-associated fibroblasts of pancreatic ductal adenocarcinoma was significantly related to invasion, metastasis, and shorter OS [22]. However, absent or decreased Nectin1 expression was found in the invading edge of uterine cervical squamous cell carcinomas compared to the center of these tumors [28]. In addition, Nectin1 expression was decreased in gastric cancer compared to normal gastric tissue and was associated with better OS [29]. Therefore, similar to FAM83H, Nectin1 might play different roles in different cancer types. In the present study, positive Nectin1 expression was significantly associated with poor prognostic factors such as higher histologic grade and higher N stage. In univariate analysis, patients with BUCs that stained positive for Nectin1 had a significantly shorter OS and RFS than patients with BUCs that were negative for Nectin1 expression. In addition, positive Nectin1 expression was an independent prognostic factor for RFS survival in BUC patients in multivariate analysis.

Another interesting finding in our study is that FAM83H expression and Nectin1 expression were significantly positively correlated in BUC samples, consistent with the association between the mRNA expression of FAM83H and Nectin1 in the cBioPortal public database. When we subdivided BUC patients according to co-expression patterns of FAM83H and Nectin1 (FAM83H+/Nectin1+, FAM83H+/Nectin1− or FAM83H−/Nectin1+, and FAM83H−/Nectin1−), we found that positive expression of both FAM83H and Nectin1 was significantly associated with higher T stage, higher histologic grade, and recurrence. Survival analysis revealed FAM83H+/Nectin1+ patient had the shortest OS and RFS of the three groups analyzed. Moreover, FAM83H+/Nectin1− or FAM83H−/Nectin1+ patients had a shorter OS and RFS than FAM83H−/Nectin1− patients. Multivariate survival analysis showed that combined expression of FAM83H and Nectin1 was an independent prognostic factor for OS and RFS in BUC patients. The results from this study indicate that FAM83H and Nectin1 are closely related to each other and play an important role in BUC progression. As mentioned above, Nectin1 is involved in cell adhesion and interacts with actin filaments [19, 20]. In a previous report, FAM83H was suggested to be a linker protein between CK-1α and keratin filaments and to be involved in the migration of cancer cells by reorganizing the keratin cytoskeleton [12]. Therefore, FAM83H and Nectin1 may interact to control the organization of cellular microfilaments such as keratin and actin filaments.

Our study has certain limitations. A major limitation is that this was a single center study and included a relatively small number of BUC patients. Therefore, additional studies with larger numbers of BUC patients from multiple centers are needed to confirm the association between FAM83H and Nectin1 expression and BUC progression. Furthermore, despite our findings indicating a possible oncogenic role for FAM83H and Nectin1 in BUC, the underlying mechanisms require clarification. In addition, future studies should determine the molecular mechanisms underlying the correlation in expression of FAM83H and Nectin1.

In conclusion, we found that FAM83H and Nectin1 expression are significantly positively associated in BUCs, and that higher expression of these proteins is significantly associated with shorter OS and RFS. Therefore, FAM83H and Nectin1 may be potential therapeutic targets in BUC patients, and the co-expression pattern of FAM83H and Nectin1 could be used as a novel prognostic indicator in BUC patients.

## Supplementary Information

**Additional file 1: Figure 1**. Relationship between mRNA expression of FAM83H and Nectin1 in bladder urothelial carcinoma. The mRNA level of FAM83H and Nectin1 showing significant correlation in TCGA, Cell 2017 database. The dataset is selected and analyzed in cBioportal database.

## Data Availability

We analyzed BUC (TCGA, Cell 2017, Weblink: https://www.cbioportal.org/study/summary?id=blca_tcga_pub_2017) dataset during current study and the dataset is available in the cBioPortal database (http://www.cbioportal.org).

## Abbreviations

TCGA: The Cancer Genome Atlas
BUC: Bladder urothelial carcinoma
HR: Hazard ratio
OS: Overall survival
RFS: Relapse-free survival
95% CI: 95% Confidence interval
