# Supplementary figures and images for: FAM83H and Nectin1 expression are related with survival and relapse of bladder urothelial carcinoma patients
Source: BMC Urol. 2021 Oct 8;21:143. doi: 10.1186/s12894-021-00908-2 (PMC8501681; doi:10.1186/s12894-021-00908-2)

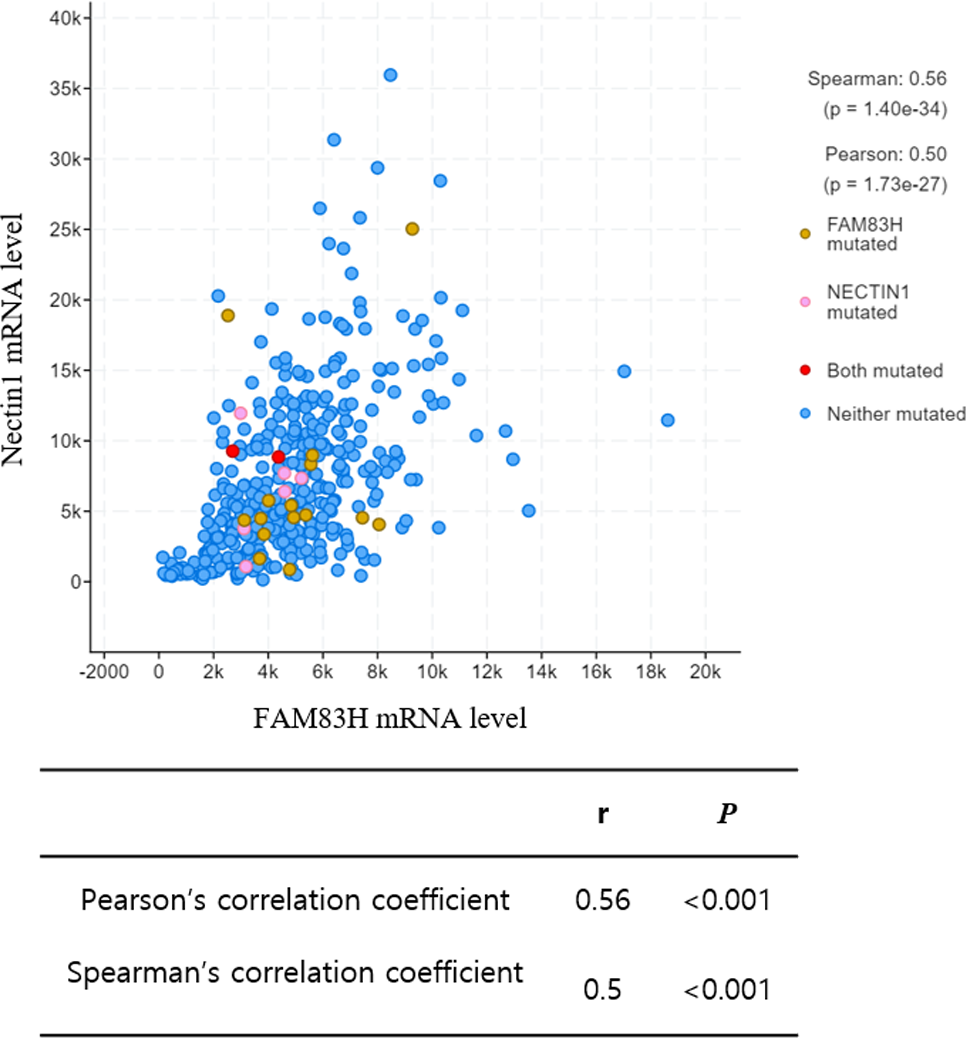

Supplement: Supplementary file 1 — Additional file 1: Figure 1. Relationship between mRNA expression of FAM83H and Nectin1 in bladder urothelial carcinoma. The mRNA level of FAM83H and Nectin1 showing significant correlation in TCGA, Cell 2017 database. The dataset is selected and analyzed in cBioportal database. [file 12894_2021_908_MOESM1_ESM.tif]
